# 3,3′-(5,5,7,12,12,14-Hexa­methyl-1,4,8,11-tetra­azacyclo­tetra­decane-1,8-diyl)di­propanonitrile methanol disolvate

**DOI:** 10.1107/S1600536810013073

**Published:** 2010-04-17

**Authors:** Cheng-Jun Hao, Yan-Hua Zhang

**Affiliations:** a College of Chemistry and Chemical Engineering, Pingdingshan University, Pingdingshan 467000, People’s Republic of China; b Department of Chemistry and Chemical Engineering, Henan University of Urban Construction, Pingdingshan 467044, People’s Republic of China

## Abstract

The asymmetric unit of the title compound, C_22_H_42_N_6_·2CH_4_O, comprises one half of a 14-membered tetra­azacyclo­tetra­decane macrocycle with cyano­ethyl substituents on one of the N atoms and a methanol solvent mol­ecule. The macrocycle lies about an inversion centre. The cyano­ethyl substituents are oriented so that the cyano groups lie over opposite faces of the central cavity of the macrocycle. The methanol solvate mol­ecules lie away from the cavity of the macrocycle and are linked to the macrocycles *via* O—H⋯N hydrogen bonds.

## Related literature

For background to macrocycles with pendant coordinating groups, see: Madeyski *et al.* (1984 ▶); Hay *et al.* (1987 ▶); Melson (1979 ▶). For a related structure, see: Roy *et al.* (2001 ▶).
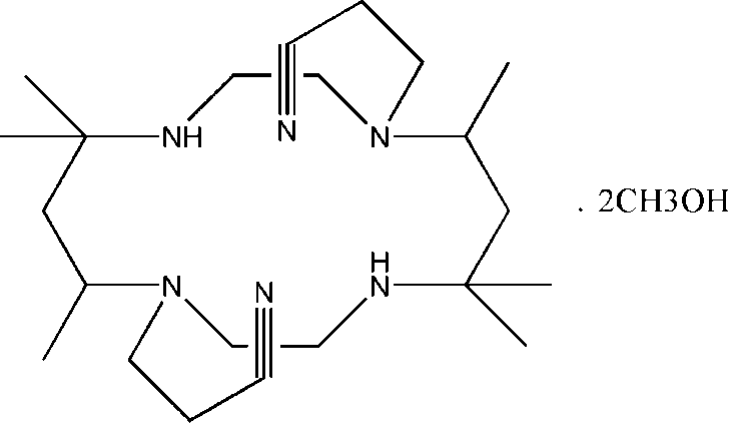


## Experimental

### 

#### Crystal data

C_22_H_42_N_6_·2CH_4_O
*M* *_r_* = 454.70
Monoclinic, 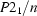

*a* = 11.8705 (16) Å
*b* = 8.4448 (11) Å
*c* = 13.4942 (18) Å
β = 94.097 (2)°
*V* = 1349.3 (3) Å^3^
*Z* = 2
Mo *K*α radiation
μ = 0.07 mm^−1^
*T* = 173 K
0.34 × 0.30 × 0.27 mm

#### Data collection

Bruker SMART 1000 CCD area-detector diffractometer
Absorption correction: multi-scan (*SADABS*; Bruker, 2004 ▶) *T*_min_ = 0.976, *T*_max_ = 0.981
10793 measured reflections
2940 independent reflections
2562 reflections with *I* > 2σ(*I*)
*R*_int_ = 0.019

#### Refinement

*R*[*F* ^2^ > 2σ(*F* ^2^)] = 0.038
*wR*(*F* ^2^) = 0.113
*S* = 1.05
2940 reflections
154 parameters
1 restraint
H atoms treated by a mixture of independent and constrained refinement
Δρ_max_ = 0.33 e Å^−3^
Δρ_min_ = −0.16 e Å^−3^

### 

Data collection: *SMART* (Bruker, 2004 ▶); cell refinement: *SAINT* (Bruker, 2004 ▶); data reduction: *SAINT*; program(s) used to solve structure: *SHELXS97* (Sheldrick, 2008 ▶); program(s) used to refine structure: *SHELXL97* (Sheldrick, 2008 ▶); molecular graphics: *SHELXTL* (Sheldrick, 2008 ▶); software used to prepare material for publication: *SHELXTL*.

## Supplementary Material

Crystal structure: contains datablocks I, global. DOI: 10.1107/S1600536810013073/sj2765sup1.cif

Structure factors: contains datablocks I. DOI: 10.1107/S1600536810013073/sj2765Isup2.hkl

Additional supplementary materials:  crystallographic information; 3D view; checkCIF report

## Figures and Tables

**Table 1:** Hydrogen-bond geometry (Å, °)

*D*—H⋯*A*	*D*—H	H⋯*A*	*D*⋯*A*	*D*—H⋯*A*
O1—H1⋯N1^i^	0.84	2.02	2.8343 (12)	162

Symmetry code: (i) 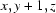.

**Fig. 1.**
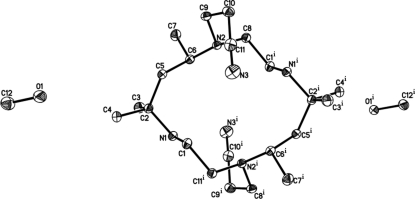
The structure of the title compound, showing the atom-numbering scheme. Non-H atoms are shown with 30% probability displacement ellipsoids (H atoms are omitted for clarity). [Symmetry code: (i) 1-x, -y, 2-z.]

## supplementary crystallographic information

### Comment

In past decades, macrocycles with pendant coordinating groups (Madeyski *et al.*, 1984; Hay *et al.*, 1987) have attracted a great deal of attention and have been studied extensively (Melson, 1979) due to the fact that their structures and properties differ markedly from those of the unsubstituted parent molecules. Recently, we have synthesized the title complex, 1,8-bis(2-Cyanoethyl)-5,5,7,12,12,14-hexamethyl-1,4,8,11-tetra- azacyclotetradecane and its structure is reported here.

The title compound, C_22_H_42_N_6_.2CH_3_OH, Fig. 1, comprises a centrosymmetric 14-membered tetra-azacyclotetradecane macrocycle with C9···C11, N3 cyanoethyl substituents on the N2 atoms and two methanol solvate molecules. These substituents are both oriented so the cyano groups lie over opposite faces of the central cavity of the macrocycle. This contrasts sharply with the situation in the structure of trans-(3*S*,5S,10*R*,12R)-1,8- bis(2-cyanoethyl)-*C*-meso-3,5,7,7,10,12,14,14-octamethyl-1,4,8,11-tetraaza- cyclotetradecane (Roy *et al.*, 2001), in which the cyanoethyl arms are directed away from the central cavity of the macrocycle. The methanol solvate molecules lie away from the cavity of the macrocycle and are linked to the macrocycles *via* O1—H1···N1 hydrogen bonds.

### Experimental

An acrylonitrile solution of *C*-meso-5,5,7,12,12,14-hexamethyl- 1,4,8,11-tetraazacyclotetradecane was heated to reflux for 6 h-10 h, The reaction mixture was cooled to room temperature and colorless crystals of the title compound were obtained by slow evaporation of the solvent at room temperature.

### Refinement

The H atom bound to N1 was located in a difference Fourier map and its coordinates and isotropic temperature factor was refined. Carbon and O bound H atoms were placed at calculated positions and were treated as riding on the parent C or O atoms with C—H = 0.98 – 1.00 Å, O—H = 0.84 Å, and with *U*_iso_(H) = 1.2 - 1.5 *U*_eq_(C, O).

### Crystal data

**Table:**

C_22_H_42_N_6_·2CH_4_O	*F*(000) = 504
*M**_r_* = 454.70	*D*_x_ = 1.119 Mg m^−^^3^
Monoclinic, *P*2_1_/*n*	Mo *K*α radiation, λ = 0.71073 Å
Hall symbol: -P 2yn	Cell parameters from 7044 reflections
*a* = 11.8705 (16) Å	θ = 2.2–27.0°
*b* = 8.4448 (11) Å	µ = 0.07 mm^−^^1^
*c* = 13.4942 (18) Å	*T* = 173 K
β = 94.097 (2)°	Block, colourless
*V* = 1349.3 (3) Å^3^	0.34 × 0.30 × 0.27 mm
*Z* = 2	

### Data collection

**Table:**

Bruker SMART 1000 CCD area-detector diffractometer	2940 independent reflections
Radiation source: fine-focus sealed tube	2562 reflections with *I* > 2σ(*I*)
graphite	*R*_int_ = 0.019
φ and ω scans	θ_max_ = 27.0°, θ_min_ = 2.2°
Absorption correction: multi-scan (*SADABS*; Bruker, 2004)	*h* = −15→15
*T*_min_ = 0.976, *T*_max_ = 0.981	*k* = −10→10
10793 measured reflections	*l* = −17→16

### Refinement

**Table:**

Refinement on *F*^2^	Primary atom site location: structure-invariant direct methods
Least-squares matrix: full	Secondary atom site location: difference Fourier map
*R*[*F*^2^ > 2σ(*F*^2^)] = 0.038	Hydrogen site location: inferred from neighbouring sites
*wR*(*F*^2^) = 0.113	H atoms treated by a mixture of independent and constrained refinement
*S* = 1.05	*w* = 1/[σ^2^(*F*_o_^2^) + (0.0618*P*)^2^ + 0.3251*P*] where *P* = (*F*_o_^2^ + 2*F*_c_^2^)/3
2940 reflections	(Δ/σ)_max_ = 0.001
154 parameters	Δρ_max_ = 0.33 e Å^−^^3^
1 restraint	Δρ_min_ = −0.16 e Å^−^^3^

### Special details

**Table:**

Geometry. All esds (except the esd in the dihedral angle between two l.s. planes) are estimated using the full covariance matrix. The cell esds are taken into account individually in the estimation of esds in distances, angles and torsion angles; correlations between esds in cell parameters are only used when they are defined by crystal symmetry. An approximate (isotropic) treatment of cell esds is used for estimating esds involving l.s. planes.
Refinement. Refinement of *F*^2^ against ALL reflections. The weighted *R*-factor wR and goodness of fit *S* are based on *F*^2^, conventional *R*-factors *R* are based on *F*, with *F* set to zero for negative *F*^2^. The threshold expression of *F*^2^ > σ(*F*^2^) is used only for calculating *R*-factors(gt) etc. and is not relevant to the choice of reflections for refinement. *R*-factors based on *F*^2^ are statistically about twice as large as those based on *F*, and *R*- factors based on ALL data will be even larger.

### Fractional atomic coordinates and isotropic or equivalent isotropic displacement parameters (Å^2^)

**Table:**

	*x*	*y*	*z*	*U*_iso_*/*U*_eq_	
C1	0.37856 (9)	0.07773 (12)	0.85259 (8)	0.0257 (2)	
H1A	0.3428	0.1592	0.8079	0.031*	
H1B	0.4073	0.1303	0.9149	0.031*	
C2	0.56583 (9)	0.10757 (12)	0.77704 (7)	0.0245 (2)	
C3	0.64934 (10)	−0.00010 (15)	0.72852 (9)	0.0338 (3)	
H3A	0.6763	−0.0821	0.7758	0.051*	
H3B	0.7135	0.0628	0.7090	0.051*	
H3C	0.6118	−0.0501	0.6695	0.051*	
C4	0.51883 (10)	0.22792 (14)	0.69961 (8)	0.0314 (3)	
H4A	0.4756	0.1722	0.6457	0.047*	
H4B	0.5815	0.2855	0.6726	0.047*	
H4C	0.4694	0.3029	0.7310	0.047*	
C5	0.62179 (8)	0.20068 (12)	0.86592 (7)	0.0239 (2)	
H5A	0.6724	0.2813	0.8397	0.029*	
H5B	0.5618	0.2580	0.8985	0.029*	
C6	0.69062 (8)	0.10371 (12)	0.94599 (7)	0.0233 (2)	
H6	0.6658	−0.0091	0.9388	0.028*	
C7	0.81748 (9)	0.10933 (15)	0.92898 (9)	0.0332 (3)	
H7A	0.8448	0.2184	0.9366	0.050*	
H7B	0.8292	0.0719	0.8618	0.050*	
H7C	0.8591	0.0413	0.9777	0.050*	
C8	0.70816 (8)	0.04726 (12)	1.12482 (8)	0.0251 (2)	
H8A	0.7295	0.1080	1.1860	0.030*	
H8B	0.7769	−0.0056	1.1036	0.030*	
C9	0.69768 (9)	0.32159 (12)	1.06682 (8)	0.0262 (2)	
H9A	0.6917	0.3826	1.0040	0.031*	
H9B	0.7775	0.3249	1.0937	0.031*	
C10	0.62306 (10)	0.39889 (13)	1.14129 (8)	0.0315 (3)	
H10A	0.6390	0.3497	1.2074	0.038*	
H10B	0.6418	0.5129	1.1472	0.038*	
C11	0.50273 (10)	0.38124 (13)	1.11086 (9)	0.0319 (3)	
C12	0.38124 (11)	0.83164 (16)	0.55435 (9)	0.0392 (3)	
H12A	0.4093	0.9349	0.5339	0.059*	
H12B	0.3183	0.7985	0.5081	0.059*	
H12C	0.4421	0.7533	0.5540	0.059*	
N1	0.47262 (7)	0.00296 (10)	0.80496 (6)	0.0234 (2)	
N2	0.66485 (7)	0.15706 (10)	1.04640 (6)	0.0220 (2)	
N3	0.40915 (9)	0.36369 (13)	1.08811 (9)	0.0448 (3)	
O1	0.34397 (8)	0.84346 (12)	0.65056 (6)	0.0406 (2)	
H1	0.3937	0.8886	0.6879	0.061*	
H1C	0.5001 (11)	−0.0715 (17)	0.8475 (10)	0.031 (3)*	

### Atomic displacement parameters (Å^2^)

**Table:**

	*U*^11^	*U*^22^	*U*^33^	*U*^12^	*U*^13^	*U*^23^
C1	0.0268 (5)	0.0226 (5)	0.0283 (5)	0.0009 (4)	0.0057 (4)	0.0009 (4)
C2	0.0254 (5)	0.0252 (5)	0.0231 (5)	−0.0026 (4)	0.0042 (4)	−0.0005 (4)
C3	0.0313 (6)	0.0390 (6)	0.0322 (6)	−0.0007 (5)	0.0093 (4)	−0.0079 (5)
C4	0.0346 (6)	0.0346 (6)	0.0248 (5)	−0.0056 (5)	0.0010 (4)	0.0050 (4)
C5	0.0241 (5)	0.0223 (5)	0.0253 (5)	−0.0023 (4)	0.0023 (4)	0.0009 (4)
C6	0.0216 (5)	0.0227 (5)	0.0259 (5)	−0.0008 (4)	0.0037 (4)	−0.0002 (4)
C7	0.0230 (5)	0.0410 (6)	0.0361 (6)	0.0027 (5)	0.0066 (4)	0.0052 (5)
C8	0.0218 (5)	0.0265 (5)	0.0268 (5)	0.0002 (4)	0.0011 (4)	0.0034 (4)
C9	0.0249 (5)	0.0229 (5)	0.0303 (5)	−0.0041 (4)	−0.0013 (4)	−0.0010 (4)
C10	0.0362 (6)	0.0258 (5)	0.0320 (6)	−0.0002 (4)	−0.0014 (4)	−0.0054 (4)
C11	0.0360 (6)	0.0233 (5)	0.0368 (6)	0.0056 (4)	0.0052 (5)	−0.0005 (4)
C12	0.0402 (7)	0.0443 (7)	0.0336 (6)	−0.0018 (5)	0.0048 (5)	−0.0082 (5)
N1	0.0236 (4)	0.0212 (4)	0.0258 (4)	−0.0011 (3)	0.0035 (3)	−0.0004 (3)
N2	0.0217 (4)	0.0205 (4)	0.0236 (4)	−0.0014 (3)	0.0008 (3)	0.0003 (3)
N3	0.0354 (6)	0.0390 (6)	0.0600 (7)	0.0072 (5)	0.0042 (5)	0.0027 (5)
O1	0.0404 (5)	0.0522 (6)	0.0294 (4)	−0.0142 (4)	0.0035 (4)	−0.0044 (4)

### Geometric parameters (Å, °)

**Table:**

C1—N1	1.4701 (13)	C7—H7B	0.9800
C1—C8^i^	1.5204 (14)	C7—H7C	0.9800
C1—H1A	0.9900	C8—N2	1.4717 (13)
C1—H1B	0.9900	C8—C1^i^	1.5204 (14)
C2—N1	1.4853 (13)	C8—H8A	0.9900
C2—C3	1.5267 (15)	C8—H8B	0.9900
C2—C4	1.5338 (15)	C9—N2	1.4639 (13)
C2—C5	1.5441 (14)	C9—C10	1.5322 (16)
C3—H3A	0.9800	C9—H9A	0.9900
C3—H3B	0.9800	C9—H9B	0.9900
C3—H3C	0.9800	C10—C11	1.4655 (16)
C4—H4A	0.9800	C10—H10A	0.9900
C4—H4B	0.9800	C10—H10B	0.9900
C4—H4C	0.9800	C11—N3	1.1408 (16)
C5—C6	1.5426 (14)	C12—O1	1.4048 (15)
C5—H5A	0.9900	C12—H12A	0.9800
C5—H5B	0.9900	C12—H12B	0.9800
C6—N2	1.4804 (13)	C12—H12C	0.9800
C6—C7	1.5401 (14)	N1—H1C	0.897 (14)
C6—H6	1.0000	O1—H1	0.8400
C7—H7A	0.9800		
			
N1—C1—C8^i^	109.62 (8)	C6—C7—H7B	109.5
N1—C1—H1A	109.7	H7A—C7—H7B	109.5
C8^i^—C1—H1A	109.7	C6—C7—H7C	109.5
N1—C1—H1B	109.7	H7A—C7—H7C	109.5
C8^i^—C1—H1B	109.7	H7B—C7—H7C	109.5
H1A—C1—H1B	108.2	N2—C8—C1^i^	112.02 (8)
N1—C2—C3	105.83 (9)	N2—C8—H8A	109.2
N1—C2—C4	109.01 (8)	C1^i^—C8—H8A	109.2
C3—C2—C4	108.56 (9)	N2—C8—H8B	109.2
N1—C2—C5	113.12 (8)	C1^i^—C8—H8B	109.2
C3—C2—C5	112.33 (9)	H8A—C8—H8B	107.9
C4—C2—C5	107.88 (8)	N2—C9—C10	111.67 (9)
C2—C3—H3A	109.5	N2—C9—H9A	109.3
C2—C3—H3B	109.5	C10—C9—H9A	109.3
H3A—C3—H3B	109.5	N2—C9—H9B	109.3
C2—C3—H3C	109.5	C10—C9—H9B	109.3
H3A—C3—H3C	109.5	H9A—C9—H9B	107.9
H3B—C3—H3C	109.5	C11—C10—C9	111.75 (9)
C2—C4—H4A	109.5	C11—C10—H10A	109.3
C2—C4—H4B	109.5	C9—C10—H10A	109.3
H4A—C4—H4B	109.5	C11—C10—H10B	109.3
C2—C4—H4C	109.5	C9—C10—H10B	109.3
H4A—C4—H4C	109.5	H10A—C10—H10B	107.9
H4B—C4—H4C	109.5	N3—C11—C10	178.26 (13)
C6—C5—C2	116.79 (8)	O1—C12—H12A	109.5
C6—C5—H5A	108.1	O1—C12—H12B	109.5
C2—C5—H5A	108.1	H12A—C12—H12B	109.5
C6—C5—H5B	108.1	O1—C12—H12C	109.5
C2—C5—H5B	108.1	H12A—C12—H12C	109.5
H5A—C5—H5B	107.3	H12B—C12—H12C	109.5
N2—C6—C7	113.28 (8)	C1—N1—C2	117.27 (8)
N2—C6—C5	110.23 (8)	C1—N1—H1C	105.9 (9)
C7—C6—C5	110.72 (8)	C2—N1—H1C	109.6 (9)
N2—C6—H6	107.4	C9—N2—C8	112.83 (8)
C7—C6—H6	107.4	C9—N2—C6	113.05 (8)
C5—C6—H6	107.4	C8—N2—C6	112.45 (8)
C6—C7—H7A	109.5	C12—O1—H1	109.5
			
N1—C2—C5—C6	68.30 (11)	C5—C2—N1—C1	57.41 (12)
C3—C2—C5—C6	−51.44 (12)	C10—C9—N2—C8	−79.38 (11)
C4—C2—C5—C6	−171.05 (8)	C10—C9—N2—C6	151.61 (9)
C2—C5—C6—N2	−136.19 (9)	C1^i^—C8—N2—C9	139.60 (9)
C2—C5—C6—C7	97.66 (11)	C1^i^—C8—N2—C6	−91.08 (10)
N2—C9—C10—C11	−52.36 (12)	C7—C6—N2—C9	61.30 (11)
C9—C10—C11—N3	80 (4)	C5—C6—N2—C9	−63.39 (10)
C8^i^—C1—N1—C2	179.81 (8)	C7—C6—N2—C8	−67.90 (11)
C3—C2—N1—C1	−179.19 (9)	C5—C6—N2—C8	167.41 (8)
C4—C2—N1—C1	−62.60 (11)		

Symmetry codes: (i) −*x*+1, −*y*, −*z*+2.

### Hydrogen-bond geometry (Å, °)

**Table:**

*D*—H···*A*	*D*—H	H···*A*	*D*···*A*	*D*—H···*A*
O1—H1···N1^ii^	0.84	2.02	2.8343 (12)	162

Symmetry codes: (ii) *x*, *y*+1, *z*.
